# Genito-Urinary and Syphilitic Diseases

**Published:** 1896-01

**Authors:** Byron H. Daggett

**Affiliations:** Buffalo. N. Y.


### Progress in Medical Science.

### GENITO-URINARY AND SYPHILITIC DISEASES.

CONDUCTED BY BYRON H. DAGGETT, M. D., Buffalo. N. Y.

### LINEAR ELECTROLYSIS.

FORT, of Paris, (*N. Y. Med. Jour.*, Nov. 16, 1895,) describes an "electrolyser" devised by himself. Fort alleges that his instrument is more effectual, more convenient and has none of the disadvantages and imperfections of the ordinary electrode. He also states that he has operated in more than 2,500 cases without serious accident and without the loss of a patient.

The electrolyser consists of a long, flexible and slender bougie, containing in the middle a projecting, smooth, dull platinum blade, connecting with a platinum wire, covered with a coating of gutta percha. The long, flexible bougie serves as a guide to prevent any false passage. The average current strength used is thirty milliampères. This method works best in small soft strictures.

### GUAIACOL AS A LOCAL ANESTHETIC.

LUCAS CHAMPIONNIÉRE and ANDRÉ (Paris) report that guaiacol applied locally acts as an anesthetic. They used solutions in sterilised olive oil, 1 part in 10 and 1 part in 20 and injected a syringeful, about 5 to 10 centigrams. These injections produced sufficient anesthesia for pulling teeth and for minor surgical operations. It requires 5 to 8 minutes for the full effect to manifest itself.

Laborde, Gilbert, Doyan and others are studying the effects of guaiacol. It is well established that this remedy produces decided local antipyretic and anesthetic results. It is stated that guaiacol is as powerful a local anesthetic as cocaine.

The abstracter uses guaiacol in castor oil, from 15 to 30 m. to the ounce, as a lubricant for urethral instruments. The castor oil is very viscid and the guaiacol is anodyne, so that the combination makes an ideal lubricant for this purpose. The guaiacol, if used too strong, will cause momentary smarting.

Eucalyptol may be added for esthetic and antiseptic purposes. We have not used it in cutting operations, but find that it takes the place of cocaine in other work.

### HYDROCELE.

Montgomery, J. M. C., reports a case of hydrocele in the female (*Therapeutic Gazette*, Nov. 16, 1895). A hydrocele in the female is rare. It was diagnosticated by exclusion and operation. There was an accumulation of fluid in the reduplication of the peritoneum over the round ligament, the remains of the canal of Nuck. This tumor presented the appearance of hydrocele in the male. It was situated over the ring which was enlarged. The sac was cut down upon and opened, when a discharge of serous fluid occurred. There was no presenting of omentum or strangulated tissue. The sac was cut away and the muscular ring closed by catgut sutures. She suffered severe pain without nausea or vomiting.

### A TEST FOR INCIPIENT DIABETES.

Noorden (*Medical Record*) states that 100 grains of grape sugar administered to the normal subject has no effect, but in the incipient diabetic and in cases having a hereditary tendency thereto, produces glycosuria. This, if experience proves it to be correct, will be a very important diagnostic procedure.

### A CASE OF URINARY SUPPRESSION.

McBurney (*Annales of Surgery*, August, 1895,) reports a case of suppression of the urine after a surgical operation. Within twenty-four hours nausea began, followed by vomiting, headache and symptoms of uremic poisoning. There was no voluntary discharge of urine and only 4 drachms were obtained by catheter in twenty-four hours. He injected a quart of salt solution into a vein of the arm. In the course of a few hours the patient voided 34 ounces of urine and steadily convalesced.

Dickinson, in the *British Medical Journal*, calls attention to the fact that patients may be aroused from diabetic coma in a few minutes by saline infusion.

### THE TREATMENT OF HYDROCELE—INJECTION OF IRRITATING SUBSTANCE IS OFTEN VERY PAINFUL.

Nicaise (*British Medical Journal*, June 5, 1895,) advises drawing off about one-third of the fluid and then passing into the sac 3 or

4 centimeters of a 1 per cent. solution of cocaine, the remaining fluid acting as menstruum. The scrotum is gently manipulated and after four or five minutes the remainder of the serous fluid is drawn off. Then inject iodine pure, or diluted one-third with water, again manipulate the scrotum four or five minutes and allow the iodine to escape. The operation is painless.

This procedure utilises a natural aseptic fluid as the excipient and the quantity of cocaine absorbed from a serous is less than from a watery solution.

---

### INFECTION OF THE URINARY TRACT.

Bastionnli (*British Medical Journal*, Oct. 26, 1895,) reports the results of his bacteriological examination of the urine in thirty-seven cases of cystitis. Microörganisms were present in every case. In twenty-five out of the thirty-seven cases, only one organism could be cultivated.

The organisms most frequently found (in twenty-one of the thirty-seven cases) were microbes belonging to the coli bacillus group, including Eberth's.

In nineteen cases there had been no catheterisation or other surgical interference.

As a result of his experience with rabbits, he states that unless pus and microörganisms are found in the urine, cystitis is not present. That by whatever pathway microörganisms enter the bladder they only induce cystitis when there is some preëxisting morbid condition of the mucosa or some impediment to the free flow of the urine.

Under such conditions the germs multiply and, insinuating themselves between the epithelial cells, cause diapedesis, suppuration and local necrosis, finally passing through the lymphatics into the circulation and system generally. These experiments indicate that microörganisms will not cause cystitis, although present in the urine, except there be some lesion of the mucosa or urinary stasis, but in combination with these conditions they are efficient causes of inflammatory action.

---

### TREATMENT OF SYPHILIS AT THE AACHEN SPRINGS.

Bogart gives an interesting report of his visit to this ancient and famous health resort (*Brooklyn Medical Journal* for Dec., 1895) These springs were known to the Romans and were made famous as a health resort by Charlemagne.

These springs belong to the class thermæ or hot water. The temperature of the waters used for therapeutic purposes is from 38° to 72° centigrade, 84° to 162° F.

These springs are rich in sulphur and alkalies and are classed as alkaline muriatic sulphur water. They contain from 22 to 28 grammes chloride of sodium, 4 to 5 grammes of sulphites and 8 to 12 grammes of carbonates to 10,000 c. cm.

The douche and vapor baths have a greater and more marked effect in stimulating tissue metabolism and secretion than do the immersion baths, especially promoting the excretion of urea and uric acid.

A bath in Aachen of 95° F., of half an hour's duration, makes the skin moist and soft and the chlorides and bicarbonates in the water free it in the simplest and most agreeable manner from adherent scales. Moreover, by the opening up of the sebaceous and sweat ducts, all obstructing masses of secretion are easily removed. Whilst these circumstances favor the increased excretion of gaseous and fluid substances, both during and after the bath, the skin is also prepared for taking up medicinal substances, which are employed with effect in the course of certain methods of treatment.—*Brissel, Aachen als Kurort.*

The Aachen treatment of syphilis is by mercurial inunction (Sigmund), in connection with the most scrupulous attention to hygiene and nutrition.

Brandis announces the following rules for the inunction treatment of syphilis: 1. The body must be adequately prepared for the absorption of the mercury and the gray ointment must always be administered carefully and in sufficient quantity. 2. The body must, during treatment, be preserved sound. 3. The inunction treatment must be carried out long enough. 4. The unguentum hydrargyri cincreum of the German pharmacopeia is used, which differs from ours by containing one-third less mercury and twice as much suet, so that the German preparation is weaker and softer, all of which renders it more suitable for inunction.

The amount of ointment at each séance is from 4 to 10 grammes (1 to 2½ drachms). The rubbing lasts twenty minutes and is done by experienced masseurs, in connection with the baths. The remedy is applied to the sides of the body, changing to other parts, according to its effects upon the skin and continued, if there be no mishaps, for eight or ten days after the syphilitic symptoms have subsided. To prevent stomatitis, camphorated chalk and acetate of alum are used to cleanse the teeth and mouth. Mercurial diarrhea is generally avoided by the daily evacuation of the bowels with the mild laxative action of the water, taken internally. Iodide of potassium is but little used.

As to duration of treatment and prognosis, Drs. Brandis and Schumacher say: so long as the healing of a syphilitic affection progresses under the action of mercury, we go on with its administration, and we combine with it other remedies when the healing process comes to a standstill. When to discontinue the inunctions depends upon individual conditions. In most cases, treatment is resumed after one to two weeks.

